# Efficacy of Yiqi Wenyang Huoxue method in treating adult bronchial asthma: A protocol for systematic review and meta-analysis of randomized controlled trials

**DOI:** 10.1097/MD.0000000000028958

**Published:** 2022-03-04

**Authors:** Qiangqiang Yu, Mengjie Bao, Tao Yu, Yuanbing Zhang, Shuangdi Xiang, Tao Li, Peng Sun, Chao Ye, Jianli Shen, Jianwei Yu, Hanrong Xue

**Affiliations:** a Affiliated Hospital, Jiangxi University of Chinese Medicine, Nanchang, China; b Jiangxi University of Chinese Medicine, Nanchang, China.

**Keywords:** adult bronchial asthma, meta-analysis, randomized controlled trials, Yiqi Wenyang Huoxue method

## Abstract

**Background:**

Bronchial asthma (BA) is a chronic inflammatory disease of the airway, which has the characteristics of recurrent attacks and difficult to cure. Glucocorticoid and bronchodilator are the primary treatment drugs for asthma. Although the treatment has made some progress, the control status is still not ideal. According to clinical reports, the Yi-qi Wen-yang Huo-xue method (YQWYHXM) of Traditional Chinese Medicine (TCM) is safe and effective in the treatment of BA, but there is not enough evidence to prove it. Based on it, we conducted this systematic evaluation.

**Methods:**

Eight databases and Clinical trial registries (Embase, Cochrane, PubMed, CNKI, CBM, Wanfang, VIP, China Clinical Trial Registry and Clinical Trails) were searched from the establishment of those until January 22, 2021 with the following terms for retrieval: BS, TCM, Chinese medicinal herb, Chinese herbal medicine and randomized controlled trial. Data analysis was performed by 2 researchers using RevMan 5.3 and SATA 16.0 separately from the Cochrane Collaboration.

**Results:**

This study will be able to provide definitive evidence to clarify all the suspicions we seek, confirming the effectiveness of YQWYHXM in the treatment of adults with BA.

**Conclusion:**

This study will prove that YQWYHXM is a safe and effective TCM adjuvant therapy for BA.

**Registration:**

Efficacy of Yiqi Wenyang Huoxue method in Treating Adult Bronchial Asthma.

**Asthma:**

A protocol for Systematic Review and Meta-Analysis of Randomized Controlled Trials. PROSPERO 2021 CRD42021256791. Available from: https://www.crd.york.ac.uk/prospero/display_record.php?ID=CRD42021256791.

## 1 Introduction

Bronchial asthma (BA) is a heterogeneous chronic airway inflammatory disease involving a variety of cells and cellular components. Recurrent wheezing, shortness of breath, with or without chest tightness and coughing are the main clinical symptoms, accompanied by high breath reactivity and variable airflow restriction. Airway remodeling will occur with the prolonged course of the disease, which is the main reason why BA is difficult to cure.

2015 Global Burden of Disease Study results showed that there were 358 million BA patients worldwide, and the prevalence rate increased by 12.6% compared to 1990,^[1]^ which was 0.7%. ∼11.9% among Asian adults (not more than 5% on average), but it is still on the rise.^[2]^ So far, BA is treated with glucocorticoids and bronchodilators, and the current status of control is still not ideal. An extensive data questionnaire survey in Europe in 2012 showed that 20.1% of BA patients achieved control, 34.8% achieved partial control, and 45.1% did not control.^[3]^

Traditional Chinese Medicine (TCM) has been involved in the whole process of China's Corona Virus Disease 2019, and it has been effectively controlled. With the promotion of TCM diagnosis and treatment technology and the further highlighting of its efficacy, people around the world are gradually paying attention to TCM. Compared with modern evidence-based western medicine, TCM is also an effective medical treatment system based on experience-based medicine, which has unique theories in defining the cause, diagnosis, prevention, and treatment of diseases. Prescription guided by the theory of TCM is composed of a variety of herbal medicines, in which the organic combination of active ingredients can treat BA in a synergistic or antagonistic way.^[4–6]^ Therefore, TCM has gradually been recognized by Western medicine as an adjuvant treatment of BS, and has even been widely prescribed as one of the clinical guidelines for treatment of BA, and has formed a special TCM diagnostic criteria for BA and a consensus among experts in diagnosis and treatment.^[7,8]^ “Xiao Bing” is the name of BA in TCM, which has long been recorded in “Huang Di Nei Jing” and “Golden Chamber Synopsis”. Wheezing in the throat, breathing difficulties, and even wheezing cannot be lying down during an asthma attack is very similar to asthma.^[9]^

According to thousands of years of TCM clinical experience, the primary pathogenesis of BA is the accumulation of phlegm in the lung, the loss of lung propaganda, and the inversion of lung-qi. The primary treatment method is resolving phlegm, dispersing lung, and relieving asthma. With the continuous innovation and development of TCM theories, modern TCM experts put forward the theory that BA is caused by Qi Yang deficiency and phlegm and blood stasis, and established the method of Yi-qi Wen-yang Huo-xue method (YQWYHXM) to treat BA,^[10]^ but there is not sufficient evidence to clarify the clinical mechanism.^[10]^ Based on it, this paper conducted a systematic evaluation of the treatment of BA by YQWYHXM to provide some evidence and reference for clinical practice.

## 2 Objective

The purpose of this systematic review and meta-analysis is to evaluate the effectiveness and safety of YQWYHXM in the treatment of BA in adults.

## 3 Methods

### 3.1 Search strategy

The retrieval include databases and Clinical trial registries such as Embase, Cochrane, PubMed, CNKI, CBM, Wanfang, VIP, China Clinical Trial Registry, and Clinical Trails. If necessary, we will try to contact the original investigator to ask for the study information we need. We will manually search the literatures related to the randomized controlled trials of the treatment of adult BA with YQWYHXM. The Key words and terms for retrieval: “bronchial asthma” OR “asthma” OR “wheezing” AND “Yiqi Wenyang Huoxue” OR “benefiting qi and warming yang” OR “benefiting qi for activating blood” OR “warming yang and promoting blood” OR “Traditional Chinese Medicine” OR “Chinese medicinal herb” OR “Chinese herbal medicine” OR “combination of Chinese traditional and western medicine” OR “TCM” AND “randomized controlled trial” OR “randomized trial” OR “controlled clinical trial” OR “clinical research” OR “randomized trial” OR “clinical trial” OR “RCT”. The search strategy of PubMed database is shown in Table 1.

**Table 1:** Search strategy used in PubMed database.

Orders	Search items
#1	((((((Asthma[Title/Abstract]) OR (Asthmas[Title/Abstract])) OR (Bronchial Asthma[Title/Abstract])) OR (xiao bing[Title/Abstract])) OR (“Asthma” [Mesh]))
#2	(((((((Traditional Chinese medicine[Title/Abstract]) OR (Chinese herbal medicine[Title/Abstract])) OR (Integrated Traditional Chinese and Western Medicine[Title/Abstract])) OR (Traditional Chinese medicine[Title/Abstract])) OR (TCM[Title/Abstract])) OR (TCM[Title/Abstract])) OR ((((((((benefiting qi warming yang activating blood[Title/Abstract]) OR (benefiting qi warming yang[Title/Abstract])) OR (benefiting qi activating blood[Title/Abstract])) OR (warming yang activating blood[Title/Abstract])) OR (((benefiting qi warming yang promoting blood[Title/Abstract]) OR (benefiting qi promoting blood[Title/Abstract])) OR (warming yang promoting blood[Title/Abstract]))) OR ((((Invigorating qi warming yang activating blood[Title/Abstract]) OR (Invigorating qi warming yang[Title/Abstract])) OR (Invigorating qi activating blood[Title/Abstract])) OR (warming yang activating blood[Title/Abstract]))) OR (((Invigorating qi warming yang promoting blood[Title/Abstract]) OR (Invigorating qi promoting blood[Title/Abstract])) OR (warming yang promoting blood[Title/Abstract]))) OR ((((Yiqi Wenyang Huoxue[Title/Abstract]) OR (Yiqi Wenyang[Title/Abstract])) OR (Yiqi Huoxue[Title/Abstract])) OR (Wenyang Huoxue[Title/Abstract])))))
#3	(((((((randomized controlled trial[Title/Abstract]) OR (randomized trial[Title/Abstract])) OR (controlled clinical trial[Title/Abstract])) OR (clinical research[Title/Abstract])) OR (randomized trial[Title/Abstract])) OR (clinical trial[Title/Abstract])) OR (RCT[Title/Abstract]))
#4	#1 AND #2 AND #3

TCM = Traditional Chinese medicine, RCT = randomized controlled trial.

### 3.2 Inclusion and exclusion criteria

#### 3.2.1 Inclusion criteria

- ***Study type***. Randomized controlled trial.
- ***Participants***. Patients over 18 years old diagnosed with typical clinical manifestations of BA, with standard reference to “Guidelines for Prevention and Treatment of Bronchial Asthma by Asthma Group of Respiratory Society of Chinese Medical Association”,^[11–14]^ there are no limitations on gender, race, asthma stage, type, course, and severity.^[11–14]^
- ***Intervention measures.*** The treatment group will be treated with YQWYHXM (decoction or injection and pill) or combined with CWM (conventional western medicine treatment included anti-infection, relieving bronchospasm, reducing phlegm and relieving asthma, oxygen inhalation, and mechanical ventilation to assist breathing, the main drugs were ambroxol, glucocorticoid, and β_2_ agonists, anticholinergics, theophylline, leukotriene antagonists, antihistamines). The control group will be treated with CWM alone. It is no time limit to the duration of treatment.
- ***Outcomes.*** According to the guidelines for clinical research on the treatment of BA with new Chinese medicine in 2002, the main outcome indicators include effective rate, changes in symptoms and signs (including changes of wheezing, cough, expectoration, chest tightness, and wheezing sound).^[15]^

Secondary outcomes will be composed of forced expiratory volume in 1 second, peak expiratory flow, forced vital capacity, forced expiratory volume in 1 second/forced vital capacity, eosinophils, IgE in serum, asthma control test, and adverse events.

#### 3.2.2 Exclusion criteria

- The Participants were younger than 18 years old and repeated in the literature.
- The Literature on other TCM therapies such as acupuncture, massage, and acupoint application.
- The Literature on animal experiments, mechanism studies, experiences, protocols, and case reports.
- The loose research literature.

### 3.3 Data extraction

**Figure 1:**
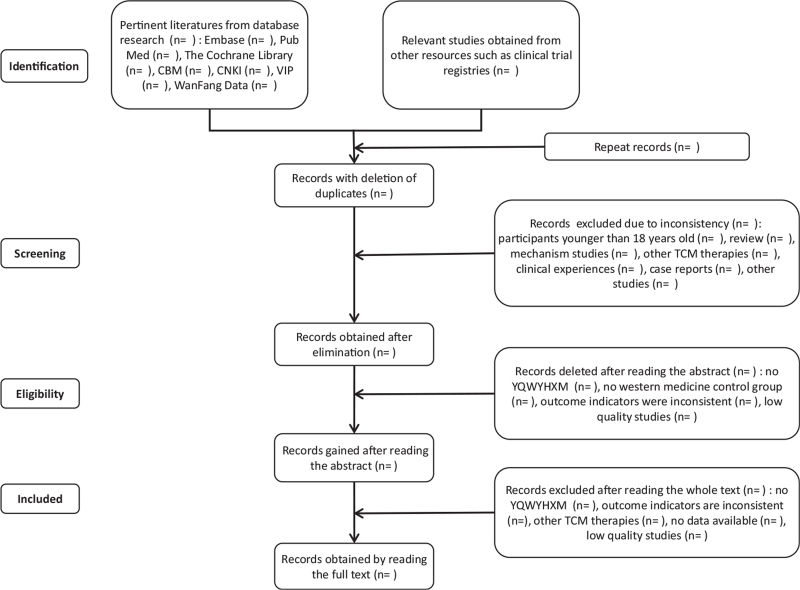
Flow chart of the study.

Two researchers will independently screen the literature according to the inclusion and exclusion criteria. In case of disagreement between the 2 researchers, the third researcher will negotiate and provide specific discussion details. The extracted data will mainly contain the eligibility of the study and the main factors of the study (study method, baseline characteristics, intervention measures, outcome indicators). If the data are incomplete, we will ask the author for relevant information (Fig. 1).

### 3.4 Data statistics

RevMan 5.3 (The Cochrane Collaboration, Software Update, Oxford,United Kingdom) and STATA 16.0 (The StataCorp LLC, Software Update, United States) provided by the Cochrane Collaboration will be used for analyzing data. Continuous variable will be measured by mean difference, standardized mean difference, and dichotomous variable will be measured by risk ratio, both of which will be expressed by 95% confidence interval. *Q* test and *I* ^*2*^ test will be used to evaluate the heterogeneity between studies. There is no heterogeneity if *P* > .1 or *I* ^*2*^ < 50%, and the fixed effect model will be used for data analysis. On the contrary, there is heterogeneity, and the random effects model will be adopted. The sensitivity analysis will be used to evaluate the impact of the included studies on the results, and Egger test will be used to assess the potential publication bias. If *P* < .05, the statistical results are considered significant.

### 3.5 Data quality assessment and statistical methods

#### 3.5.1 Risk of bias assessment

The 6 elements of randomized controlled trial of quality assessment provided by the Cochrane Collaboration will be adopted, including random method, allocation scheme concealment, blind method, data integrity, selectively reporting research results, and bias of other sources.^[16]^ Each item will be evaluated at 3 levels: low risk, high risk, and uncertainty.

#### 3.5.2 Heterogeneity assessment

The heterogeneity will be assessed by a χ^2^ test with a significance level of P < .1. Computing the *I* ^2^ statistic is a quantitative method for assessing inconsistency in heterogeneity between studies. A value of 0% indicates no heterogeneity, while an *I* ^2^ value ≥50% indicates a significant level of heterogeneity. However, heterogeneity is only assessed when certain conditions are met when performing a meta-analysis.

#### 3.5.3 Assessment of publication bias

A funnel plot will be used to detect publication bias if the results include more than 10 articles. In addition, we will use Egger test to detect the symmetry of the funnel plot.

#### 3.5.4 Subgroup analysis

We will perform subgroup analysis of the data according to factors such as patient age, gender, course of disease, and interventions to clarify the efficacy and safety of YQWYHXM in the treatment of adult BA.

#### 3.5.5 Quality of evidence

We will adopt the GRADE approach to evaluate the degree of the evidence to make our results more evidence-based and credible. This study had been registered with PROSPERO 2021 CRD42021256791 (https://www.crd.york.ac.uk/prospero/display_record.php?ID=CRD42021256791). This study will be strictly performed in accordance with the Preferred Reporting Items for Systematic Review and Meta-Analysis Protocols.^[17]^

### 3.6 Ethical approval

Since this study is based on a systematic review of published studies and will not involve patient privacy and right to know, it does not require ethics committee review and approval.

## 4 Discussion

Bronchial asthma is a chronic airway inflammatory disease with recurrent and incurable characteristics. Although some progress has been made in the conventional western medicine treatment based on glucocorticoids (or) and bronchodilators, there are many adverse reactions, and the patients’ compliance with long-term medication is poor, resulting in an unsatisfactory control status. In China, YQWYHXM has been used to treat bronchial asthma. Animal experimental studies have shown that this method can regulate the imbalance of T lymphocytes and inhibit asthmatic airway inflammation.^[18–21]^ Although clinical randomized controlled trials have also shown that this method is effective in the treatment of asthma, there is no systematic evaluation to confirm its effectiveness, and it has not been recognized internationally. Therefore, we intend to conduct a comprehensive systematic review and meta-analysis to provide the latest evidence for the treatment of BA with YQWYHXM.

## Author contributions

**Conceptualization:** Qiangqiang Yu, Yuanbing Zhang, Jianwei Yu, Hanrong Xue.

**Data curation:** Mengjie Bao, Tao Yu, Tao Li, Peng Sun.

**Software:** Shuangdi Xiang, Chao Ye, Jianli Shen.

**Supervision:** Jianwei Yu, Hanrong Xue.

**Writing – original draft:** Qiangqiang Yu, Tao Yu, Jianwei Yu.

**Writing – review & editing:** Qiangqiang Yu, Tao Yu, Hanrong Xue.
